# Psychological Well-Being, Multiple Identities, and Discrimination Among First and Second Generation Immigrant Muslims

**DOI:** 10.5964/ejop.v14i1.1434

**Published:** 2018-03-12

**Authors:** Cristina Giuliani, Semira Tagliabue, Camillo Regalia

**Affiliations:** a Department of Psychology, Università Cattolica del Sacro Cuore, Milano, Italy; b Department of Psychology, Università Cattolica del Sacro Cuore, Brescia, Italy; Department of Psychology, Webster University Geneva, Geneva, Switzerland; University of Neuchâtel, Neuchâtel, Switzerland

**Keywords:** discrimination, Muslims, immigration, identity, first and second generation, psychological well-being

## Abstract

Given the growing number of Muslim immigrants in Western countries, there is a need for research focusing on their psychological well-being and correlates. The present study investigated whether perceived discrimination is associated with depression and satisfaction with migration through the mediating role of several identity dimensions (ethnic, national, and religious) among 204 first and second generation adult Muslim immigrants living in Italy. They participated in structured interviews, and a multi-group path analysis model was conducted using Mplus. While the impact of perceived discrimination on psychological well-being was modest for first generation Muslims, in the case of second generation Muslims perceived discrimination was directly associated with lower psychological well-being (higher depression and lower satisfaction with the migration decision) and indirectly associated with satisfaction with migration through the mediation of national and religious identity. The higher the levels of discrimination that second generation Muslims perceived, the weaker their national (host country) identity and the greater their religious identification. In turn, national and religious identities were associated with respectively higher and lower levels of satisfaction regarding their migration decision. The findings showed clear differences between first and second generation immigrant groups, revealing that perceived discrimination represents an obstacle to integration processes more for second generation immigrants than for first generations.

Immigration and acculturation (that is, the process of adapting to a majority or new cultural context) are stressful experiences that can have positive or negative consequences on the psychological well-being of immigrants and ethnic minority youth (Berry, 1997). Research carried out in the last decades, mostly in countries with long and established tradition of immigration (e.g., the United States, Canada), has extensively documented the complex and multifaceted struggles and challenges faced by immigrants and their descendants. Focusing on specific ethnic minorities living in the West, most studies have shown how immigrants’ and ethnic minorities’ well-being is linked to several acculturation-specific risk and protective factors that distinguish immigrants’ experience, in accordance with their migration history, national origin, and resettlement experiences (Berry, 1997; Miconi, Moscardino, Altoè, & Salcuni, in press; Phinney, Horenczyk, Liebkind, & Vedder, 2001). Among these factors, discrimination and racism have been found to negatively affect psychological well-being among ethnic minority groups (e.g., Asian, Latino, and Black Americans; Brittian et al., 2015; Lee, 2003; Utsey, Ponterotto, Reynolds, & Cancelli, 2000).

Little is known about immigrants from Muslim-majority countries who live in the West (Sirin & Fine, 2008). Like other minority groups, Muslim communities are frequently the targets of negative prejudice; notably, they face growing discrimination as a consequence of the 9/11 attacks and their aftermath, which has generated an atmosphere of fear and suspicion towards them (Pew Research Center, 2016). More recently, concern about Muslim communities has become a prominent issue in the media and public discourse, which have emphasized the role played by young Muslim generations—born and educated in Western countries—in violent actions (riots, terrorist attacks in Europe). Indeed, in the last decade, researchers began to explore the impact of current shifting socio-political contexts on the integration process of Muslim immigrants in countries with long and established tradition of immigration (e.g., the United States, Canada, the United Kingdom) (Bhatia & Ram, 2009; Sirin & Fine, 2007; Thomas & Sanderson, 2011). Also, in many European countries, where Muslim immigrants and their descendants constitute a large community today (e.g., Germany, France, the Netherlands; Pew Research Center, 2010), the issue of Muslim immigrants’ integration and psychological well-being has become urgent. This issue is crucial not only for the first generation of Muslim immigrants, who have directly experienced migration, but also for second generation youth, who were born and are growing up in Western countries and are primarily exposed to the host cultural environment during their formative years (Berry & Sabatier, 2010; Sirin & Fine, 2008; Stevens, Pels, Vollebergh, & Crijnen, 2004). Second generation Muslims need to negotiate multiple issues pertaining to identity processes, and need to combine feelings of belonging to their ethnic and religious community with those to the host country. Furthermore, they face these challenges within a social and political context characterized by terrorist events and growing religious discrimination (Allen & Nielsen, 2002; Sirin & Balsano, 2007). For these reasons, concerns about the mental health and psychological well-being of Muslim youth increase (Berry, Phinney, Sam, & Vedder, 2006; Heim, Hunter, & Jones, 2011; Van Geel & Vedder, 2010).

Empirical studies examining the psychological well-being of Muslims living in Europe (mainly Moroccans and Turks) are scant and exploratory in nature. A large number of factors have been proposed as being associated with the psychological well-being of Muslims. These include socio-demographic variables (e.g., gender, age, age at the time of immigration, length of stay in the host country, generation of immigration), post-migration variables (e.g., acculturation factors, religious identity), and social contextual variables (e.g., perceived discrimination). The present study tested a model investigating how these variables are associated with the psychological well-being of first and second generation immigrants.

## Perceived Discrimination, Identity, and Well-Being

The discrimination that immigrants perceive in their country of settlement is one of the major stressors for immigrants, and negatively impacts their acculturation and psychological adjustment, as widely recognized in the literature (Brittian et al., 2015; Utsey et al., 2000). For immigrants, perceptions of being discriminated—that is, being unfairly and differentially treated—may be conveyed in many forms based on their ethnicity, race, and religious membership (Brittian et al., 2015; Lee, 2003). A strong relationship between perceived ethnic and racial discrimination and poorer mental health outcomes (including lower self-esteem and higher rates of depression, anxiety, and psychosomatic complaints) has also been widely documented among second generation and minority youths in a variety of countries (Berry et al., 2006; Fisher, Wallace, & Fenton, 2000; Lee, 2003; Utsey et al., 2000). More recently, in a current climate marked by growing fear related to Islamic radicalization and terrorism (Alba, 2005; Allen & Nielsen, 2002), some preliminary studies have explored the link between perceived ethnic and religious discrimination and psychological well-being among first generation (Heim et al., 2011) and second generation Muslims living in the West (Berry & Sabatier, 2010; Vedder, Sam, & Liebkind, 2007). However, further evidence in the context of Europe is needed.

Several studies support a relationship between perceived discrimination and different dimensions of identity processes. For Muslims living in Western Europe, acculturation includes not only the negotiation of competing heritage and mainstream host cultural orientations, but also issues of religious diversity within European societies that are historically Christian, highly secularized, and increasingly hostile to the presence of Muslims in Western societies (Duderija, 2008; Güngör, Fleischmann, & Phalet, 2011; Verkuyten, 2007; Verkuyten, Thijs, & Stevens, 2012). Indeed, although immigration and acculturation studies have mostly neglected religious identity dimensions (Sheikh, 2007), a growing number of studies about Muslim immigrants have revealed that Muslim identification and religiosity (practices, beliefs, values) are salient components of daily life for Muslim immigrant communities (Duderija, 2008; Hussain & Bagguley, 2005; Thomas & Sanderson, 2011). The majority of Muslim immigrants in Europe shows high or the highest scores on measures of Muslim identification (Thomas & Sanderson, 2011; Verkuyten, 2007), hence confirming the centrality of this identity dimension and its meaning as an identity marker.

Many authors have also noted that the demarcation between ethnic identity and religion is “blurred”; religious and ethnic identities are positively intertwined for first generations and, even more, for second generation immigrants (Duderija, 2008; Güngör et al., 2011; Maliepaard, Lubbers, & Gijsberts, 2010; Verkuyten & Yildiz, 2007), whereas the relationship between religious and national identification is unclear. Some studies have reported that among Muslim youth residing in Europe, the highest level of religious identification implies a low level of national (host country) identification (Alba, 2005; Crul & Doomernik, 2003; Heim et al., 2011; Maliepaard et al., 2010; Verkuyten et al., 2012; Verkuyten & Yildiz, 2007).

The task of coping with multiple cultural systems of reference is intensified within societies where immigrants experience prejudice and rejection for their religious and ethnic affiliations (Brewer, 2010), as it has been happening in recent years in European societies characterized by the rise of Islamophobia (Pew Research Center, 2016). High levels of perceived discrimination seem to explain why “biculturalism” (that is, a combination of strong heritage and strong mainstream cultural orientations) is not a feasible choice for Muslims in Europe, forcing them to defend and valorize their in-group identification (Fleischmann, Phalet, & Klein, 2011; Heim et al., 2011). Perceived discrimination is associated with both a strong or exclusive ethnic group attachment and a weakening of the ties with the host cultural context (Berry & Sabatier, 2010; Fleischmann et al., 2011; Sabatier 2008; Sirin et al., 2008; Vedder et al., 2007) among second generation Muslims. Furthermore, the view of Islam as a threat to “Western” identity and mainstream values produces a defensive intensification of Muslim in-group identification (Verkuyten & Yildiz, 2007).

## Immigrant Muslims’ Identities and Psychological Well-Being

Perceived discrimination is associated with both Muslims’ well-being and identity, and identity processes, in turn, are associated with well-being (Berry et al., 2006; Berry & Sabatier, 2010). Indeed, multiple dimensions of Muslims' identity and how they change during the acculturation process strongly affect the psychological well-being of these immigrants and second generation youth, as widely documented in acculturation studies (Berry et al., 2006).

The complexity and multidimensional nature of Muslims’ identity processes in post-migration settings has been documented (Britto, 2008; Giuliani, Olivari, & Alfieri, 2017; Giuliani & Tagliabue, 2015; Verkuyten, 2007; Verkuyten & Martinovic, 2012; Verkuyten et al., 2012), but studies have rarely considered simultaneously how multiple and interrelated aspects of Muslims’ identities (e.g., ethnic, national, and religious) impact their psychological well-being (Brewer, 2010). Notably, religious identity has been rarely included in studies that explore heritage (ethnic) or mainstream (national) cultural orientations (Verkuyten et al., 2012; Verkuyten & Yildiz, 2007), yet it should be included to understand the extent to which different dimensions of identity are likely to differently affect well-being. Indeed, the bulk of research on post-migration identity processes and adaptation outcomes stems from the two-dimensional model of acculturation based on the study of Berry (1997). According to this model, more positive adaptation outcomes imply that immigrants are able to navigate between their own heritage culture and the host culture and to integrate them. Hence, two independent dimensions are considered crucial in the process of identity construction: ethnic acculturation attitude (i.e., maintenance of own heritage cultural identity) and national attitude (i.e., contacts in larger society, adoption of cultural values of the nation of settlement) (Arends-Tóth & van de Vijver, 2004; Berry & Sabatier, 2010; Donà & Berry, 1994; Goforth, Oka, Leong, & Denis, 2014).

Similarly to what has been found in studies on different groups of immigrants and ethnic minorities (Berry et al., 2006), research on Muslim immigrants has widely explored national (host country) and ethnic (country of origin) components of identity (Phinney et al., 2001) and their role in adjustment. A combination of strong ethnic and national attitudes—leading to better psychological adjustment—has been documented in studies conducted in countries with a long history of immigration and of promoting “multiculturalism” as national policy, such as the United States, Canada, Australia, New Zealand, and the United Kingdom (Nguyen & Benet-Martínez, 2013). In these countries, which Berry and colleagues (2006) called “settler societies” for their colonial origin and multiculturalism, Muslim immigrants (as with all immigrants) have more opportunities to successfully combine their own culture and the host culture (Berry & Sabatier, 2010; Britto & Amer, 2007; Sirin & Fine, 2007, 2008; Stuart, Ward, & Adam, 2010; Zaal, Salah, & Fine, 2007).

Conversely, European studies about Muslim immigrants—in particular, Moroccans and Turks living in Europe (the Netherlands, Belgium, the United Kingdom, France, Germany, Finland)—document greater difficulties experienced by Muslims in their acculturation process and adaptation outcomes. Ethnic and national attitudes are not or are negatively correlated (Heim et al., 2011; Ouarasse & van de Vijver, 2005; Sabatier, 2008) among first and second generation Muslims. Furthermore, ethnic identity prevails over national identity (Sabatier, 2008; Verkuyten et al., 2012). Therefore, immigrants rarely achieve an integrated or bicultural identity and more frequently adopt a separation strategy—that is, a path characterized by a high value placed on the wish to retain their own cultural heritage and, simultaneously, little interest in interacting with and adopting the host society culture (Arends-Tóth & van de Vijver, 2004; Berry & Sabatier, 2010; Crul & Doomernik, 2003; Heim et al., 2011; Stevens et al., 2004; Vedder et al., 2007; Verkuyten, 2007). The link between separation strategy and well-being outcomes still remains largely unexplored in European studies about Muslim communities. According to the bidimensional acculturation model (Nguyen & Benet-Martínez, 2013), separation is a less adaptive strategy in comparison with biculturalism or integration (i.e., high ethnic and high national cultural preferences), whereas evidence from research among highly devalued minority groups (e.g., Latino, African, and Asian minorities living in the United States) has shown that a strong or exclusive ethnic identity may represent an alternative means of protecting identity and enhancing psychological well-being (Branscombe, Schmitt, & Harvey, 1999; Brittian et al., 2015; Phinney, 1990; Umaña-Taylor, 2011). For example, the rejection-identification model (Branscombe et al., 1999) suggests that perceived discrimination and rejection lead individuals to intensify their in-group identifications, which in turn protects their psychological well-being.

Additionally, research shows that second generation Muslims are more likely to struggle with combining loyalty to their cultural heritage and host society values than their adult counterparts. Hence, they not only retain a strong ethnic identity but are also more willing—in comparison to their adult counterparts—to adopt values and practices of the host country (Stevens et al., 2004). A recurring issue is that they can be unable to face the task of reconciling these two cultural universes. In Stevens and colleagues’ (2004) study, for instance, Muslim Moroccan girls experienced major conflict between loyalty to their family and community heritage and external pressures like the gender models offered in Western host countries.

Studies that focus on ethnic (country of origin) and national (host country) cultural orientations (without creating typologies of strategies) reveal different associations with well-being. In particular, a strong orientation towards ethnic culture among Muslims leads to positive psychological well-being and has a protective effect on health outcomes, in particular fewer psychological problems (depression, anxiety, psychosomatic symptoms) and better life satisfaction and self-esteem (Heim et al., 2011; Ouarasse & van de Vijver, 2005; Vedder et al., 2007). On the other hand, national identity (i.e., adoption of host country culture, participation in larger society) seems more linked to socio-cultural aspects of adjustment such as school and work success as well as successful participation in the host society (Heim et al., 2011; Ouarasse & van de Vijver, 2005).

Studies have also considered religious identity (Amer & Hovey, 2007; Friedman & Saroglou, 2010; Jasperse, Ward, & Jose, 2012; Stuart et al., 2010). While religiosity has a positive role in the acculturation process and adjustment to the host society for immigrant populations (Abu-Rayya & Abu-Rayya, 2009; Harker, 2001), little is known about the link between religious identity and adjustment among Muslim immigrants. In North American and New Zealand studies, religiosity (strong Muslim identity, high engagement in religious practices) is predictive of fewer psychological symptoms and higher life satisfaction among first and second generation Muslim immigrants (Amer & Hovey, 2007; Jasperse et al., 2012; Stuart et al., 2010). In European studies, this link is rarely investigated, and preliminary findings show a more complicated and negative relationship between religiosity and well-being among Muslims living in Belgium, where they suffer from religious discrimination and stigmatization (Friedman & Saroglou, 2010).

No study has simultaneously investigated the mediator role of ethnic, national, and religious identities in the relationship between perceived discrimination and psychological adjustment (Mossakowski, 2003; Phinney, 1990). For example, Heim and colleagues (2011), in a recent study on minority youth living in the United Kingdom (including a Pakistani subgroup), showed that perceived discrimination influences physical and psychological well-being via multiple mediators, like minority (ethnic) and majority (national) identities. They suggested that discrimination intensifies ethnic identification, which in turn negatively influences perceived stress and positively influences psychological well-being. Moreover, discrimination weakens national identification, which in turn negatively influences perceived social capital and physical well-being. No consideration of the role of religious identity was included in the study (Heim et al., 2011).

Moreover, the literature on psychological well-being and adaptation among first and second generation Muslim immigrants often focuses on positive (subjective life satisfaction, self-esteem, good mental health) and negative (behaviour problems, anxiety, and depression) dimensions but only rarely on well-being dimensions specifically related to the migration experience (Williams, Jobes, & Gilchrist, 1986). In this regard, the current study used two indices of psychological well-being: depression and satisfaction with migration decision. The former has been widely used in previous studies on discrimination, while the latter represents a migration-specific aspect of psychological well-being that has rarely been used in research on immigrants (Williams et al., 1986). Satisfaction with migration decision measures the degree of satisfaction related to the choice of leaving the country of origin (whether the decision was carried out by themselves or made by their parents or family of origin), the willingness to repeat the choice, or conversely, the desire to return to the country of origin. Since it expresses a reconfirmation or a rejection of the family migration project, it is an interesting index of psychological well-being for comparing the experience of the first and second generations of immigrants.

The majority of the above-mentioned studies involves second generation Muslims (e.g., Berry & Sabatier, 2010; Crul & Doomernik, 2003) yet it does not distinguish the generational status of immigrants (Verkuyten & Yildiz, 2007). There is a lack of studies comparing post-migration experiences of first and second generations in the considered domains (acculturation, identity processes, perceived discrimination).

Based on these considerations, the aim of the present research was to investigate the mediating role not only of ethnic and national but also of religious identity in the association between discrimination and two aspects of psychological well-being (i.e., depression and satisfaction with the migration decision), by comparing first and second generations.

## Aim

**Figure 1:**
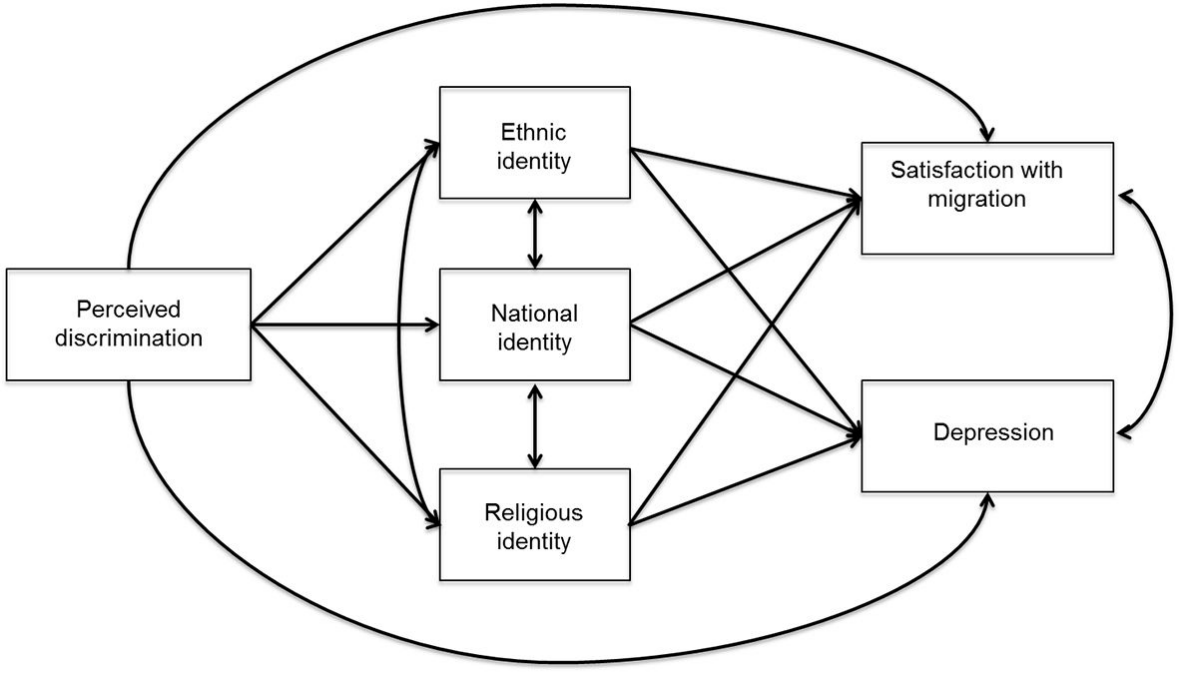
Mediational model.

Evidence in the literature has shown that discrimination and multiple dimensions of identity processes influence psychological well-being. Moreover, a recent study revealed the mediator role of ethnic and national identity (Heim et al., 2011). In the present study, we aimed to test a model in which perceived discrimination affects Muslims’ psychological well-being (depression and satisfaction with migration) through the mediation of religious, ethnic, and national dimensions of identity processes in first and second generation Muslim immigrants. Following the widely used bidimensional cultural model of Berry (1997), we explored two independent dimensions of identity: attitudes and identities towards the “Italian culture” (national identity) and attitudes and identities towards their heritage cultural background (ethnic identity). Furthermore, we investigated religious identity by considering two of its facets (Islam identification and religious practices). The model is presented in Figure 1.

We go beyond existing research in three ways. First, we focused simultaneously on multiple intertwined dimensions of identity processes (ethnic, national, and religious). Second, we examined psychological adaptation not only with depression but also with a specific and new index focused on the assessment of migration experience, such as the level of satisfaction with migration. Third, we investigated the direct and indirect role of perceived discrimination comparing first and second generation Muslim immigrants.

## Method

### Participants

The participants were 204 adult Muslim immigrants who originated from many Muslim-majority countries and lived in the highly urbanized northern part of Italy. The age of the participants ranged from 18 to 72 years, with a mean age of 36.6 (*SD* = 12.1). Among them, 59.8% were first generation immigrants (range: 35–72 years; *M* = 45.6 years, *SD* = 5.77; 59% males, 41% females), and 40.2% were second generation immigrants (range: 18–34 years; *M* = 23.3 years, *SD* = 4.18; 42.7% males, 57.3% females). First generation immigrants were defined as those born in a non-Western country who came to Italy when they were young adults. Using a broad operational definition of second generation, we included in the second generation immigrant group either Italy-born children of foreign parents or foreign-born children who were brought to Italy at the age of 11 or younger (Rumbaut & Portes, 2001).

A majority of the participants were married (59.1%; the majority belonged to the first generation, among whom 89.3% were married), while 34% were single (the majority belonged to the second generation, among whom 82.9% were single), 5.9% were separated or divorced, and 1% were widowed. In terms of education, the modal educational level was high school or less (71% for first generation; 91% for second generation). Only 28% of the first generation and 8.6% of the second generation participants had a college or advanced degree.

Among participants, 67.5% came from North African states (mainly from Morocco and Egypt); the others had a different non-Western origin, such as Sub-Saharan Africa (11.8%), South Asia (11.8%), and Eastern Europe (9.9%). On average, first-generation immigrants came to Italy when they were 28.5 years old (*SD* = 5.30); among the second generation participants, 28% were born in Italy, and 72% arrived in Italy before they turned 11 years old (*M* = 4.33; *SD* = 3.86). The average length of residence in Italy was 17.8 years (range: 3–44 years; *SD* = 6.26): 17.1 years (range: 3–44 years; *SD* = 6.86) among first-generation and 18.9 (range: 7–34 years; *SD* = 5.08) years among second generation immigrants. A majority of the participants (91.8%) reported being Sunni Muslim, which is the most represented Muslim group in Italy (Fondazione Ismu, 2017).

### Procedure

Participants were recruited with the support of ethnic and religious groups and organizations located in the Milan metropolitan area. Additionally, we employed “snowballing” methods for recruiting participants who met the criteria for participation (i.e., self-identified as Muslim, first and second generation, 18 years and older) and approached organizations, groups, and individuals. Data were collected by means of a questionnaire (pretested by the first author and presented in Italian) in a one-on-one interview by trained multilingual interviewers from February to December 2015. Before collecting the data, the questionnaire was evaluated by a multidisciplinary team that included the researchers and linguistic/cultural/religious experts from the International Oasis Foundation (www.oasiscenter.eu) in order to verify the religious and cultural appropriateness and meaningfulness of each item for Muslim participants.

The interviewer read the questions and filled out the questionnaire. Only participants who were foreign-born and not fluent in Italian, were assisted by trained multilingual interviewers. The interview took 60 min on average to complete and took place in public spaces (cafes, libraries, associations, schools) selected by the respondents.

The participants were informed that their participation was voluntary and that their responses would be confidential. Written informed consent was obtained. The University Ethical Committee approved the research protocol, which fulfilled the ethical standards of the Italian Psychology Association (AIP, Associazione Italiana di Psicologia).

### Measures

In addition to a socio-demographic information sheet, the questionnaire contained measures of ethnic, national and religious identity, perceived discrimination, and psychological well-being.

*Ethnic and national identity*. Two dimensions of identity were assessed through a reduced version of the Acculturation Attitudes Scale (AAS) of Berry and Sabatier (2010). Specifically, 11 items reflected identification with “heritage culture” (ethnic identity), and six items assessed identification with the “mainstream Italian culture” (national identity) related to various domains (language, social networks, values, emotion, cultural transmission). Sample items included: “I think that it is important that [*heritage culture*] be maintained across generations”, “I like to attend [*own ethnic group*] parties”, “I think that [*ethnic*] parents should make an effort for their children to develop ties with [*own ethnic group*] people outside the house” for ethnic identity; “I like to attend to *Italian* parties”, “I want to adopt the *Italian* way of life”, and “I think that parents should make an effort for their children to develop relationships with *Italian* society” for national identity. The participants were asked to indicate their level of agreement on each item on a scale ranging from 1 (*totally disagree*) to 5 (*totally agree*). The Acculturation Attitudes Scale was translated from French into Italian, and an independent back translation into French was performed to check the accuracy of the translation. The literature has established good validity and reliability for this measure (Berry & Sabatier, 2010). In the current study, we performed an exploratory factor analysis using principal axis factoring, extracted one factor explaining 42.46% of variance (factor loadings for the ethnic dimension ranged between .48 and .84, whereas the factor loadings for national dimension ranged between .38 and .80). The coefficient alphas were .85 for ethnic identity and .81 for national identity.

*Religious identity.* Religious identity was measured with one pictorial item for religious identification and several for religious practices. *Religious in-group identification* was assessed with a single pictorial item derived from the Inclusion of In-Group in the Self measure (IIS, Tropp & Wright, 2001), which consists of seven Venn-like diagrams, with pairs of circles varying in their degree of overlap, and the respondents were asked to select the pair of circles that best represented their level of identification with a given in-group (in this case, with Islam). Responses to the IIS ranged from 1 (*two separate circles/no overlap*) to 7 (*high degree of overlap*). Tropp and Wright’s study (2001) established good validity for this one single-item measure. *Religious practices* was assessed with six items, selected from Güngör and colleagues (2011). The items measured the participants’ observance of religious rules and rites, including dietary practices (e.g., eating halal food), worship (e.g., saying prayers), religious transmission (explicit, e.g., the teaching of religious beliefs, and implicit routes of religious transmission, e.g., parental modeling of religious behaviours). The measure included items such as “How often did you fast during the last Ramadan?”, “How often do you say daily prayer?”, and “Did you attend Koran lessons as a child?”. A 5-point rating scale (from 1 = *none* to 5 = *regular practice*) indicated the frequency with which the participants engaged in specific practices. The items were translated from English into Italian, and an independent back translation into English was performed to check the accuracy of the translation. Good validity and reliability of the measure have been established by Güngör and colleagues (2011). An exploratory factor analysis, using principal axis factoring, extracted one factor explaining 58.66% of variance (factor loadings ranging between .64 and .87). An overall religious index (termed Religious Identity), including both religious in-group identification and behavioural components of Muslim identity (α = .89), was created.

*Perceived discrimination.* Perceived discrimination was measured by two items derived from Fleischmann and colleagues (2011). The items addressed personal experience of unfair treatment, offensive words, or hostility due to religion or background more generally, and were rated on a 5-point scale ranging from 1 (*never*) to 5 (*almost daily*). The items were “Have you ever experienced hostility or unfair treatment because of your origin or background?” and “Have you ever experienced hostility or unfair treatment because of your religion?”. The items were translated from English into Italian, and an independent back translation into English was performed to check the accuracy of the translation. Good validity and reliability of this measure have been established (Fleischmann et al., 2011). In our study, the items formed a reliable scale (*r*(204) = .57, *p* < .001).

*Psychological well-being.* Positive (satisfaction with migration decision) and negative (depressive symptoms) indicators of psychological well-being were used. *Satisfaction with migration decision* was measured by three items created ad hoc. The items addressed subjective satisfaction with the choice for the participants to leave their country of origin (whether the decision was carried out by themselves or made by their family of origin) and willingness to repeat the migration choice. An examples of the items included: “If I could go back, I would make the same choice”. The response options ranged from 1 (*totally disagree*) to 5 (*totally agree*). For the present study, we conducted an exploratory factor analysis using principal axis factoring and asking for one factor; the analysis extracted one factor explaining 46.76% of the variance (factor loadings ranging between .50 and .92). The reliability was sufficient (α = .64). *Depression.* Depression was measured by six items from the Center for Epidemiologic Studies Depression Scale (CES-D, Radloff, 1977; Italian version, Fava, 1983) in order to assess depressive symptoms (e.g., “I was bothered by things that don’t usually bother me”; “I felt that everything I did was an effort”). The responses ranged from 0 (*rarely or none of the time to less than 1 day)* to 3 (*most or all of the time to 5–7 days).* A good validity and reliability of this measure have been confirmed for Italian translation (Fava, 1983). For the present study, we conducted an exploratory factor analysis using principal axis factoring and asking for one factor; the analysis extracted one factor explaining 49.70% of the variance (factor loadings ranging between .51 and .86). The coefficient alpha for this was is .85.

## Results

The means and intercorrelations among the measures are presented in Table 1.

**Table 1:** Means, Standard Deviations, and Intercorrelations Among Measures (First and Second Generation Immigrant Muslims)

Variable	1st generation		2nd generation		*t*	*df*	*p*	1	2	3	4	5	6
	*M*	*SD*	*M*	*SD*									
1. Perceived discrimination	2.15	0.95	2.10	0.81	-0.39	202	.689	—	.14	.06	.16	.10	-.20*
2. Ethnic identity	3.61	0.62	3.30	0.68	-3.27	202	.001	.28*	—	.39**	.45**	.11	-.14
3. National identity	3.16	0.72	3.39	0.71	2.22	202	.027	-.38**	-.14	—	.07	.25**	.17
4. Religious identity	3.72	1.06	2.79	1.07	-6.05	201	.000	.29**	.75***	-.26*	—	.04	-.22*
5. Depression	1.05	0.63	1.07	0.64	0.17	201	.868	.36**	.36**	-.02	.34**	—	-.19*
6. Satisfaction with migration	3.02	0.96	3.38	0.75	2.68	180	.008	-.38**	-.31**	.38**	-.37**	-.23	—

*Note.* Correlations for first generation Muslims are above diagonal, correlations for second generation Muslims are below diagonal.

*p < .05. **p < .01. ***p < .001.

### Mediational Model

We tested, using Mplus 7.1 (Muthén & Muthén, 1998–2010), a multi-group path-analysis model, in which discrimination is connected with depression and satisfaction with migration through the mediation of ethnic and national identity, as well as religious identification. The model is a saturated one because we tested both direct and indirect effects of perceived discrimination. We first ran a baseline model, in which the same model was simultaneously tested in the first and second generations, but the path estimates were allowed to be different across the two groups. Then, we tested a constrained multi-group model in which all of the paths were constrained to be equal; this second model was significantly different from the baseline one, Δχ^2^(16) = 40.588, *p* < .05; CFI = .868; RMSEA = .123 (90% CI [.076, .170]).

**Figure 2:**
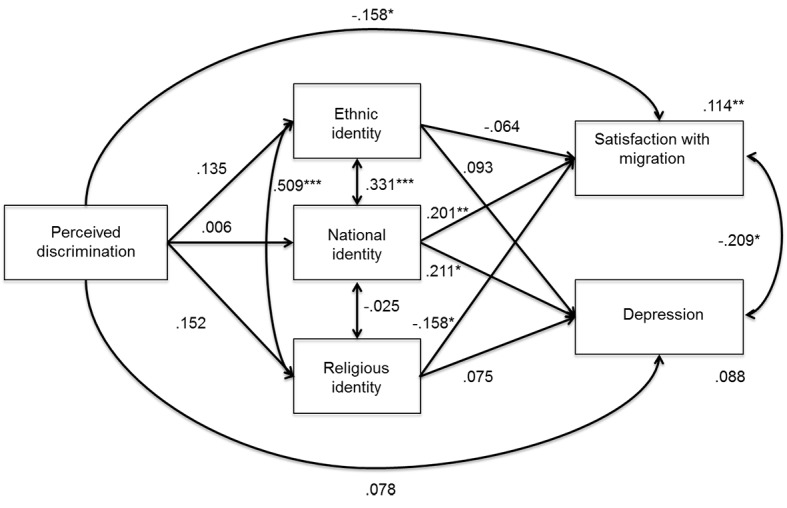
Multigroup mediational model: First generation Muslims. *p < .05. **p < .01. ***p < .001.

**Figure 3 f3:**
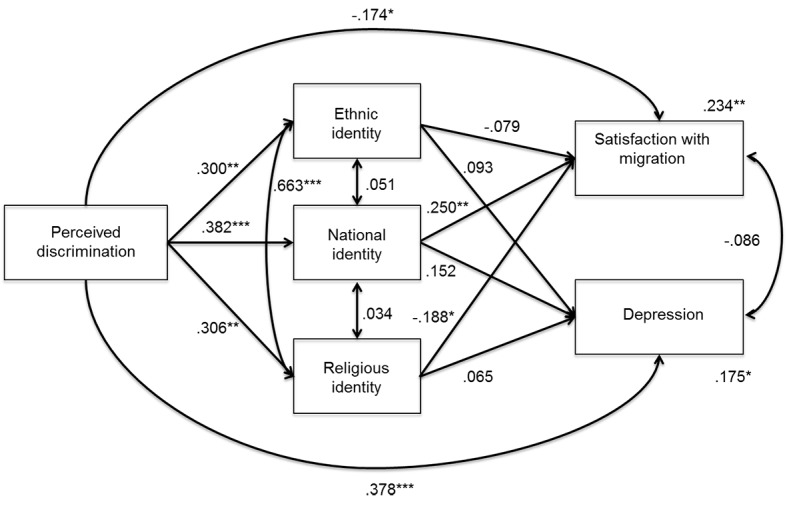
Multigroup mediational model: Second generation Muslims. *p < .05. **p < .01. ***p < .001.

The inspection of paths in the baseline model revealed that some, but not all, paths were similar between the two groups. Thus, we tested a partial invariant multi-group model in which we constrained those paths to be equal (the direct effects of ethnic and religious identity on depression, all of the paths between the independent and mediator variables and satisfaction with migration, and the covariances between religious identification and national and ethnic identity). This model was not significantly different from the baseline multi-group model, Δχ^2^(8) = 13.495, *p* = .096; CFI = .971; RMSEA = .082 (90% CI [.000, .156]). Figures 2 and 3 [Fig f3] show the standardized paths in the first and second generation models.

Among first generation immigrants, discrimination was directly and negatively associated only with satisfaction with migration, whereas it was not significantly linked with any aspects of identity. For second generation immigrants, discrimination was directly associated with depression (positively associated) and satisfaction with migration (negatively associated), and indirectly associated with satisfaction with migration through the mediation of national and religious identities. Indeed, discrimination was negatively associated with national identity, which, in turn, was positively associated with satisfaction with migration; also, discrimination was positively associated with religious identity, which in turn was negatively associated with satisfaction. Moreover, discrimination was positively associated with ethnic identity, although no mediation was found for that dimension of identity. The variance explained was larger for second generation than for first generation immigrants, whose depression variance was not significantly explained by the other variables. It is also noteworthy that depression and satisfaction with migration were significantly and negatively associated with each other only among first generation immigrants.

Despite the absence of mediation, among first generation immigrants, national identity was significantly and positively associated with both satisfaction with migration and depression, and religious identity was significantly and negatively associated with satisfaction with migration.

Finally, among the first and second generation immigrants, the covariance between ethnic and religious identity was significant and positive, whereas the other covariances among ethnic, national, and religious identity were not significant, with the exception of the one between ethnic and national identity among the first generation.

## Discussion

The present study aimed to test a mediational model in which perceived discrimination was hypothesized to affect psychological adjustment directly and through the mediation of multiple dimensions of identity processes, comparing first and second generation Muslim immigrants.

The findings show clear differences between first and second generation immigrants and only give support to the posited mediational model for second generation immigrants. Second generation immigrants who perceived high levels of discrimination seemed to have weakened national identity perception and increased religious identification, as previously suggested (Fleischmann et al., 2011; Heim et al., 2011; Verkuyten & Yildiz, 2007), which in turn is associated with respectively higher and lower levels of satisfaction about their migration decision. Hence, national and religious identities are likely to mediate the effect of discrimination on satisfaction with migration but not on depression.

Direct links between discrimination and both depression and satisfaction with migration decision were also found for both generations. In particular, second generation immigrants who felt discriminated against were more depressed, and participants in both generations who felt discriminated against were less satisfied with their decision to immigrate.

Considering both indirect and direct paths, we found that the percentage of variance in psychological well-being explained by both discrimination and identity was higher and significant for second generation and lower for first generation immigrants. These results are in line with many previous European studies about Muslim immigrants (Berry & Sabatier, 2010; Vedder et al., 2007). As suggested by Heim and colleagues (2011), discrimination seems to be a stronger obstacle for second generation immigrants than for first generations, making integration into the new society more difficult. This finding is also consistent with previous research showing the role played by generational status (first and second generation) on the post-migration experience of Asian and Latino minority groups (Inman, 2006; Kaduvettoor-Davidson & Inman, 2013; Liu & Suyemoto, 2016; Wiley, Deaux, & Hagelskamp, 2012).

Indeed, although we did not directly test the climate of suspicion in Italy, we can speculate that analogously with other stigmatized ethnic minorities. The experience of being discriminated against within the countries where they were born or grew up may be more challenging for second generation Muslim youth than for first generation people. Previous research seems to support this interpretation about the different perceptions of discrimination across generations (Inman, 2006; Kaduvettoor-Davidson & Inman, 2013; Liu & Suyemoto, 2016; Wiley et al., 2012). Firstly, second generation individuals would have greater exposure to discrimination experiences than first-generation individuals as a result of having more opportunities to interact in socialization contexts (school and workplaces, with peers and adults, such as teachers). They grow up and spend their formative years within host societies, where they are a religious/ethnic minority and where religious and ethnic markers are emphasized (Kaduvettoor-Davidson & Inman, 2013). Higher linguistic competences among the second generation are likely to facilitate and increase opportunities for interactions within the host society and make the second generation more aware of the subtleties of unfairness and discrimination (Kaduvettoor-Davidson & Inman, 2013). Furthermore, the maintenance of a stronger connection with homeland cultural aspects (language, habits and traditional customs and values, social networks) among the first generation is likely to buffer their exposure to negative and harmful experiences in the host country (Inman, 2006; Kaduvettoor-Davidson & Inman, 2013). On the contrary, second generation youth are deeply engaged in developmental processes of identity construction, which lead to more conflicts between external and family pressures, making them more vulnerable to external pressure (Stuart et al., 2010). Lastly, as suggested by Liu and Suyemoto (2016), whereas first generation adults born outside the host country attribute discrimination experiences to their immigrant status, second generation individuals (but also 1.5 generation individuals who grew up in the host society) tend to consider themselves as members of the host society, and perceive discrimination as a result of their distinctiveness (linked to ethnicity, race, or religiosity). Thus, the second generation is likely to attribute their negative experience in the host society to societal barriers and systemic rejection, and they are less optimistic and more disillusioned about the future than the first generation (Wiley et al., 2012).

Direct effects have also been found between discrimination and identity processes, but only for second generation immigrants. In fact, among the second generation, discrimination is positively associated with ethnic and religious identities and negatively associated with national identity. Hence, when they feel discriminated against, they tend to have both intensified links with their religious and ethnic systems of reference and a weakened link with the mainstream culture. These findings are in line with several European studies regarding young Muslims living in Europe (Berry et al., 2006; Berry & Sabatier, 2010; Sabatier, 2008; Sirin et al., 2008; Vedder et al., 2007), which confirm the role played by discrimination on identity processes. As expected, discrimination seems to produce defensive behaviour and strengthen bonds with the ethnic and religious in-group (Fleischmann et al., 2011; Heim et al., 2011; Verkuyten & Yildiz, 2007). However, Verkuyten (2007) suggested distinguishing two different processes: in fact, a weak national identity could reflect a low level of identification with the host group (that is, people resisted identifying themselves with the mainstream culture) or a national de-identification from the host group (that is, people do not want to belong to the host society). According to Verkuyten (2007), only in the second case do immigrants reject and distance themselves from the host group and are able to develop an “oppositional identity” (p. 354), whereas in the former case, people can be indifferent towards the other culture but have a sense of belonging and commitment to the new society.

Finally, direct links have been found between identity dimensions and the two aspects of well-being that were investigated. National identity is the only aspect of identity that is significantly linked with better psychological well-being for second generation (and partially for first generation) immigrants, whereas ethnic and religious identities were not. In particular, when second generation Muslims identify themselves as Italian, they are satisfied about their migration decision. For first generation Muslims, high national identity seems to generate ambivalent feelings: high satisfaction with migration but also higher levels of depression and anxiety. This may be seen as a counterintuitive finding, and more research is needed to understand which moderating variables could explain this link. For instance, there is empirical evidence showing that ambivalent feelings are especially present among second generation Muslim women (Gennari, Giuliani, & Accordini, 2017; Stevens et al., 2004). Cultural pressures seem to be especially consistent for young Muslim women who are caught between loyalty to heritage cultural orientations and attraction to Western gender models, placing them in a more vulnerable position within Western contexts conducive to ambivalent outcomes on well-being. They would be depressed because they feel constrained to a traditional role that is difficult to break: as the main custodians of traditional and heritage values, it would be difficult for them to be completely satisfied with “Western” values supporting a more autonomous self-construal. Another explanation could be related to the extent to which first generation Muslims are satisfied with their decision, while contextual circumstances—and perhaps the limited recognition as nationals that they receive from others—might cause a sense of *otherness,* and thus causing them anxiety or making them feel depressed. Future studies could shed light on this issue and show which speculation is more adequate.

Overall, these findings confirm previous studies about Muslim immigrants in Europe, particularly those on second generation immigrants, in which national identity was generally found to be positively connected to psycho-social well-being (Heim et al., 2011; Ouarasse & van de Vijver, 2005).

Conversely, our study failed to confirm the buffering role played by ethnic and religious identity on well-being (Heim et al., 2011; Ouarasse & van de Vijver, 2005; Vedder et al., 2007). In fact, no significant association between ethnic identity and the two indices of well-being (depression and satisfaction with migration decision) was found for either first or second generation immigrants. Furthermore, unlike previous research suggesting that religiosity is positively related to well-being (Abu-Rayya & Abu-Rayya, 2009; Harker, 2001; Jasperse et al., 2012), in our study, religious identity (including measures of identification with Islam and engagement in practices) did not positively impact either well-being outcome. Rather, a negative link between religious identity and satisfaction with migration was found among both generations: the more important the religious dimension was for them, the more dissatisfied they were with their choice to leave their country of origin and to live in another context. Our findings are consistent with Friedman and Saroglou’s (2010) Belgian study, which show that the relationship between religiosity and well-being is complex within societies where religiosity represents an important aspect of stigmatization of Muslims (Pew Research Center, 2016). Among Muslims facing consistent stigmatization in current Western intergroup contexts (like Belgium but also like many other European countries), religiosity has been associated with decreased self-esteem and increased depression through the intervening variables of perceived religious intolerance and feelings of anger towards the host culture (Friedman & Saroglou, 2010, p. 192).

Finally, it is interesting to note that ethnic and religious identity dimensions are strongly positively intertwined for first- and, even more, for second generation immigrants. This result is in line with studies confirming that the demarcation between ethnic and religious components of identity is subtle and tends to become even more confused for second generation immigrants (Güngör et al., 2011; Maliepaard et al., 2010; Verkuyten, 2007; Verkuyten et al., 2012; Verkuyten & Yildiz, 2007). However, unlike European studies, in which national and religious identities are negatively correlated or incompatible orientations (Heim et al., 2011; Ouarasse & van de Vijver, 2005; Sabatier, 2008), in our work, national identity was not correlated with the religious dimension (for both generations) and was weakly positively correlated with ethnic components (only for the first generation). In other words, our data suggest that ethnic and religious identities highly overlap and are not perceived as being incompatible with national identity. Hence, it seems Muslims’ in-group affiliations do not occur at the expense of identification with the host culture.

Three aspects distinguishing Italy from other European countries in which those issues were investigated could explain those differences. First is the specific and weak nature of Italian secularism in comparison with those of other European countries like France or the Netherlands (Diotallevi, 1999; Kosmin & Keysar, 2009; Roy, 2007): in Italy, the Catholic Church, activism by ecclesiastical organizations in many sectors (school, sport, social assistance, education, health, etc.), and religious values remain strong and exert influence on the public and political spheres. Religiosity and the role of religion in social and political domains could be perceived as an element of proximity between Muslims and Catholics. Second, the history of Muslim immigration is more recent in Italy than in other European countries, and their density is lower. Third, Italy has been less directly affected by Muslim terrorism, at least until now, keeping antagonism between the autochthon and Muslim communities lower than in other parts of Europe.

In sum, it is conceivable that the important role of religion for Italians along with the lower perception of threat linked to the Muslim presence can contribute to explaining why Muslims’ religious and ethnic belonging are not perceived as being opposite to Italian cultural values. As pointed out by Vedder and colleagues (2007), little is known about post-resettlement factors associated with acculturation processes and adaptation. In this regard, comparative studies within Europe are needed to explore the complexity of the relationship between Muslim immigrants’ acculturation experience and different countries of settlement.

### Limitations and Conclusions

The study presents some limitations. The first issue concerns the cross-sectional nature of the design, thus limiting the possibilities for testing causal links among variables. For example, although many empirical studies support the causal link between perceived discrimination and intensification of ethnic identity, evidence also suggests that a strong ethnic/religious identification may increase perceptions of discrimination. Longitudinal research will be important in future studies to establish directional influences (Phinney, 1990).

The second limitation concerns the choice and recruitment of participants. Sampling biases derived from the participant recruitment (a convenience sample, based on voluntary participation from a population living in the highly urbanized northern part of Italy) may reduce the representativeness of the Italian social reality and the demographic context. Furthermore, Muslims constitute a very heterogeneous group of people: the label Muslim used for selecting participants does not capture the diversity of national origins among immigrant Muslims living in Italy, and our data did not allow us to explore the role played by the context of origin on acculturation processes (Phinney et al., 2001). Future studies could directly focus on the largest Muslim immigrant communities in Italy (that is, Moroccans, Albanians, Egyptians, Bangladeshis, Pakistanis, and Tunisians; Fondazione Ismu, 2017; ISTAT, 2016). Moreover, the small sample of second generation participants in the current study did not allow us to explore gender differences. As documented (Sirin & Fine, 2008; Stevens et al., 2004), future studies should also explore the second generation experience among men and women. “Culture” and “Islam” set different standards and norms for males and females, thus influencing their migration experience and well-being in the host country.

Finally, additional limitations to note concern the instruments used to assess the main constructs investigated in the current study. Perceived discrimination, identity, and well-being are multidimensional constructs. With respect to discrimination, recent studies have highlighted how several types of ethnic/religious discrimination experiences have different impacts on well-being (Brittian et al., 2015). Notably, it would be useful in future studies about Muslims to consider perceived ethnic group discrimination (e.g., denigration of one’s groups as a whole) in addition to the widely used interpersonal (individual) perceived discrimination. Furthermore, we need to consider the multidimensionality of the concept and measurement of identity dimensions. Each of the identity dimensions explored in the current study (ethnic, national, and religious identity) includes different aspects and operationalizations (e.g., sense of belonging, centrality, identification, adoption of cultural practices and values, citizenship) that are rarely investigated in the same study. We adopted a cultural framework to define identities; nevertheless, future studies should include more facets of identity processes, in line with recent research interests, such as the study of citizenship identities and their relationship with national identity (Hussain & Bagguley, 2005; Thomas & Sanderson, 2011). Lastly, we only considered the psychological well-being of Muslims, whereas researchers recommend investigating several aspects of well-being. No indicators of socio-cultural well-being (e.g., school success, work performance, behavioural problems) have been included in the current study in addition to psychological well-being, and both are indeed relevant outcomes of the acculturation process, as already documented (Searle & Ward, 1990).

Notwithstanding these limitations, the present study is the first in Italy to underline the negative role played by perceived discrimination among second generation Muslims. Complex relationships among perceived discrimination, identity dimensions, and well-being have been shown, mainly for second-generation Muslims and less for first generation Muslims. In line with previously cited European literature, the former seem to face more challenging tasks in facing discrimination and reconciling multiple identity dimensions. An innovative aspect of the study was the evaluation of immigrants’ psychological well-being using an index of satisfaction that was specifically related to their migration decision, which is rare in the literature. Our findings about that variable were particularly interesting for second generation immigrants: when they felt discriminated against and “less Italian”, they were unsatisfied and rejected the migration decision, which had not been made by themselves but by others (their parents or their family of origin). Thus, the second generation’s psychological well-being is strictly connected to the personal meaning that these youth give to their family migration project: they need to personally reconfirm the migration project carried out in the past by their parents to experience well-being. Future studies need to further explore these issues to understand and support integration processes among Muslim generations in the face of increasing discrimination.
